# Optimizing Culture Conditions by Statistical Approach to Enhance Production of Pectinase from* Bacillus* sp. Y1

**DOI:** 10.1155/2019/8146948

**Published:** 2019-02-20

**Authors:** Fenfen Guo, Xuezhi Li, Jian Zhao, Guanxi Li, Peike Gao, Xiaolong Han

**Affiliations:** ^1^State Key Laboratory of Microbial Technology, Shandong University, Qingdao City 266237, China; ^2^School of Life Sciences, Qufu Normal University, Qufu, Shandong 273165, China; ^3^Department of Life Science and Engineering, Jining University, Qufu, Shandong 273155, China

## Abstract

It was found that* Bacillus *sp. Y1 could secrete alkaline pectinase with suitable enzyme system for powerful and fast degumming of ramie fiber. In this study, the medium components and fermentation conditions were optimized by some statistical methods including mixture design, fractional factorial design, central composite design and response surface methodology, and single factor method for enhancing the alkaline pectinase production. The optimized conditions for pectinase production were that the culture was shaken at 34°C for 60 h in 50 mL of medium containing 10.5% (w/v) carbon source (consisting of 3.8% starch, 4.2% wheat bran, and 2.5% sucrose), 0.37% (NH_4_)_2_SO_4_, 0.3% MgSO_4_, and 0.1% Tween-80, with initial pH 8.2 and inoculation amount of 1.3 mL (with the OD600 of the seed medium about 5.77). Using the optimizing conditions, the activities of polygalacturonate lyase (PGL) and polygalacturonase (PG) in fermentation liquor were increased to 2.00-fold and 3.44-fold, respectively, and the fermentation time shortened 12 hours (from 72 h to 60 h), which showed good application potential in degumming of ramie.

## 1. Introduction

Pectinases are enzymes that contribute to pectin degradation through various mechanisms. Based on mode of action and preferred substrate, these enzymes can be briefly classified as pectin methyl esterases (PME, E.C 3.1.1.11) and pectin acetyl esterase (PAE, E.C 3.1.1.6) which removes methoxyl and acetyl residues from pectin to produce polygalacturonic acid, and the other subclass of homogalacturonan degrading group of enzymes which are broadly termed depolymerases breaking the *α*-1,4 linkages either by hydrolysis, i.e., polygalacturonases (PG, E.C 3.2.1.15) or via transelimination mechanism, namely, polygalacturonate lyases (PGL, E.C 4.2.2.2) and pectin lyases (PNL, E.C 4.2.2.10) [1]. Pectinase produced by various microorganisms are useful in a number of industrial applications and can be apparently classified into acidic and alkaline pectinases according to their pH requirement for optimum enzymatic activity. Acidic pectinases from fungal sources (*Aspergillus niger*,* Penicillium notatum, *and* Botrytis cinerea*) are used in the fruit juice industries and wine making. Alkaline pectinases, mostly from bacterial sources especially* Bacillus* sp., are mainly used in the degumming and retting of fiber crops, pretreatment of pectic wastewater from fruit juice industries, coffee and tea leaf fermentation, oil extraction, virus purification, etc. [1, 2]. Decorticated ramie fibers contain 20% to 35% gum, which consists of pectin, hemicellulose, lignin, and so on. The pectinase is commonly considered as the key enzyme during enzymatic degumming of ramie fibers, especially PGL, and hemicellulases may also play a role for enzymatic degumming, but lignin-degrading enzymes have little effect on the degumming process [3–5]. Studies on the optimization of culture conditions and media, fermentation methods (solid-state, submerged fermentation), and screening of strains have been done to enhance PGL and PG production [6–12]. The gene that encodes PGL (*pels*) has also been expressed in recombinant* Pichia pastoris* GS115 [13] and homologously overexpressed in* Bacillus subtilis *[14] to obtain high production of the PGL. However, studies showed that high PGL or PG activity in pectinase does not mean the removal of more pectin [3, 15], which showed that a suitable degumming enzyme system was also very important for promoting enzymatic degumming.

Our previous study had proved that the alkaline pectinase produced from wild-type* Bacillus *sp. Y1 had more suitable enzyme system with powerful and fast degumming ability and suitable characteristics for degumming of ramie fibers, especially the high tolerance for H_2_O_2_ [16]. What is more, the synergistic action was also found between the alkaline pectinase and H_2_O_2_ on the degumming and bleaching of ramie fibers. The protease component in the crude pectinase was further substantiated to be an important factor in degumming process, and synergistic action between protease and pectinase was also found during degumming. These studies showed that the alkaline pectinase of* Bacillus *sp. Y1 had unique advantages and deserved further study. To further enhance pectinase production, some statistical design methods, for example, the mixture design, fractional factorial design (FFD), central composite design (CCD), and response surface methodology (RSM), were employed to optimize the media compositions, such as the ratio between three carbon source components, suitable media components, and its concentrations in this study. Statistical methods are effortless, time-saving, and considering interactions among variables. The single factor method was also applied to optimize culture conditions including culture temperature and inoculation amount.

## 2. Materials and Methods

### 2.1. Microorganism and Media

*Bacillus *sp. Y1 was previously isolated from a soil sample collected from some bast fiber degumming factory, China, and conserved on nutrient agar slants at 4°C in our laboratory.

The seed culture medium consists of 10 g/L glucose, 5 g/L peptone, 5 g/L NaCl, 10 g/L K_2_HPO_4_, 0.5 g/L MgSO_4_·7H_2_O, and 5 g/L pectin. The pH of the seed medium was adjusted to 8.0. The strain was incubated on a shaker at 100 rpm and 37°C for 8 h and transferred to the fermentation medium to produce pectinase.

During optimization, the submerged fermentation was conducted in 300 mL Erlenmeyer flasks by taking 50 mL of medium containing 54 g/L wheat bran, 42 g/L starch, 3 g/L (NH_4_)_2_SO_4_, 2 g/L MgSO_4_·7H_2_O, 1 g/L Na_2_CO_3_, 1 g/L Tween-80, and initial pH 8.5 on a swing shaker (100 rpm) at 34°C for 72 h, according to previous experiments. After fermentation, the supernatant of the culture broth was obtained through centrifugation to determine the enzyme activity.

### 2.2. Optimization of Culture Conditions

Based on preliminary work in our laboratory, the effects of culture temperature and inoculation amount on pectinase production were studied using single factor experiments. Strain Y1 was cultured through submerged fermentation (SmF) in 300 mL shaking flask at 30, 34, and 37°C for 60 and 72 h, with inoculum sizes of 0.65, 1.30, 2.17, and 3.03 mL of the seed medium (with the OD600 about 5.77) volume per 50 mL of fermentation medium, respectively. The optimum conditions were selected by comparing the enzymes activity of the fermentation liquor.

### 2.3. Optimization of Culture Media Components by Experimental Designs

#### 2.3.1. Mixture Design of the Carbon Source

Mixture design was performed using design expert software “Minitab” (Minitab Inc.). All experimental designs were randomized. Mixture design strategy, which uses a limited number of experiments to study multicomponent systems, is based on statistical analysis. The experimental region can be represented by a regular simplex. This region is a triangle because the sum of the component proportions is united. The experimental region is explored at a point of composition that corresponds to an ordered arrangement. The mixture design method assumes that pectinase production is a function of its carbon source composition. Three carbon source components are present in the culture media, namely, starch, wheat bran, and sucrose, which comprises 7% (w/v) of the total. These three components were mixed according to a mixture design matrix that prescribes ten experiments (Table 1). The response surfaces for PGL production were obtained using the Minitab statistical software. The quadratic model of response Y and independent variables were expressed as follows:

(1) Y=βaXa+βbXb+βcXc+βabXaXb+βacXaXc+βbcXbXc

where X_a_ is starch; X_b_ is wheat bran; X_c_ is sucrose; Y is the predicted response; *β*_a_, *β*_b_, and *β*_c_ are the linear coefficients; *β*_ab_, *β*_ac_, and *β*_bc_ are the interactive coefficients.

#### 2.3.2. Fractional Factorial Design

Fractional factorial design (FFD) was used to screen the factors that significantly affect pectinase production from the carbon source (x_1_), (NH_4_)_2_SO_4_ (x_2_), K_2_HPO_4_ (x_3_), MgSO_4_ (x_4_), Tween-80 (x_5_), and initial pH (x_6_). The experimental design included 19 experiments with three central points. In FFD, the statistical design was expressed as coded values for convenience, in which –1, 0, and +1 stand for the low, high, and middle levels, respectively. Based on the results of previous experiments in our laboratory, the factors and levels of the factors in FFD are shown in Table 2.

#### 2.3.3. Central Composite Design

The central composite design (CCD), which uses the response surface methodology (RSM), was adopted to further optimize the significant variables selected using the FFD method. These variables included the carbon source (x_1_), (NH_4_)_2_SO_4_ (x_2_), and initial pH (x_6_). The MgSO_4_ and Tween-80 concentrations in the media were 3 and 1 g/L, respectively. A 2^3^ factorial rotatable central composite design with six star points (*α* = 1.68) and six replicates at the center points (n_0_ = 6), which led to 20 experiments (Table 4), was used to optimize the levels for pectinase production. The quadratic model of response Y (for each parameter) and independent variables were expressed as follows:

(2) Y=β0+β1X1+β2X2+β6X6+β11X12+β22X22+β66X62+β12X1X2+β16X1X6+β26X2X6

where Y is the predicted response; *β*_0_ is the intercept; *β*_1_, *β*_2_, and *β*_6_ are the linear coefficients; *β*_11_, *β*_22_, and *β*_66_ are the squared coefficients; and *β*_12_, *β*_16_, and *β*_26_ are the interactive coefficients.

For the mixture design and CCD, the Minitab statistical software was used to obtain the optimal values for these factors and “STATISTICA” software was used to generate the response surfaces.

All experiments were carried out in triplicate and mean values were applied.

### 2.4. Measurement of Enzyme Activity

The PGL activity was determined by measuring the absorbance of unsaturated bonds in the product at 235 nm. The reaction mixture contained 2 mL of 0.2% (w/v) polygalacturonic acid (Sigma Chemical Co. type P3889) in 0.05 M glycine–NaOH buffer (containing 0.44 mM CaCl_2_) at pH 9.6 and 20 *μ*L of the diluted enzyme solution. The reaction mixture was incubated at 45°C for 15 min. Then, the reaction was terminated by adding 3 mL of 0.03 M phosphoric acid. The product was checked using a spectrophotometer (Shimadzu, UV-2550). One enzyme unit (U) was defined as the amount of enzyme that produces 1 *μ*mol of unsaturated galacturonic acid per min with a molar extinction coefficient of 4600 [13].

The PG activity assay was performed by incubating 0.5 mL of the suitably diluted enzyme with 1 mL of 0.5% pectin (Sigma Chemical Co. type P9135) in 0.05 M glycine–NaOH buffer (pH 9.6) at 55°C for 30 min. PG activity was determined by measuring the amount of d-galacturonic acid liberated by the enzyme action using a spectrophotometer [17]. One unit (U) was defined as the amount of enzyme required to release 1 *μ*mol of galacturonic acid from polygalacturonic acid per min under the assay conditions.

## 3. Results

### 3.1. Optimization of Culture Temperature and Inoculation Amount

**Figure 1:**
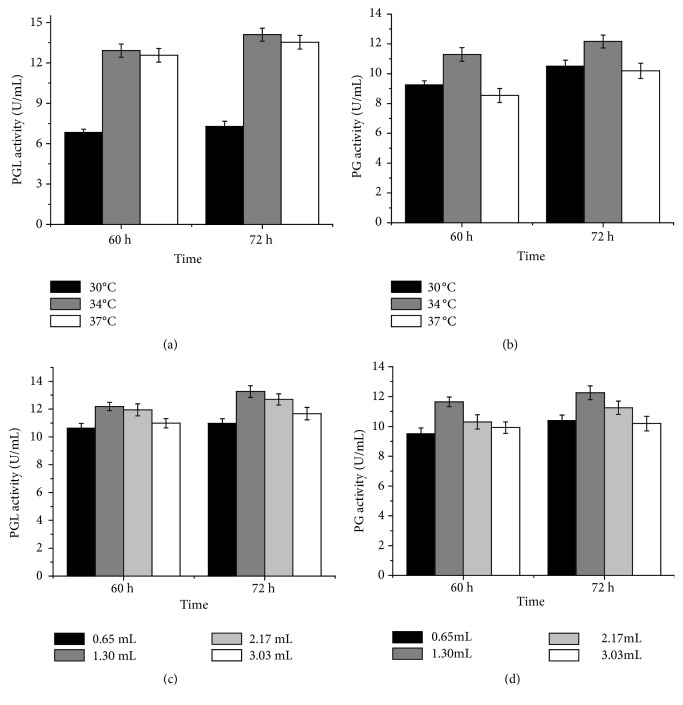
The effects of culture temperature and inoculation amount on PGL (a,c) and PG (b,d) production at 60 and 72 h.

Figure 1 showed the effects of culture temperature and inoculation amount on pectinase production at 60 and 72 h of fermentation time, respectively. It was demonstrated that the optimum temperature and inoculation amount for production of pectinase from* Bacillus *sp. Y1 was 34°C and 1.3 mL (with the OD600 of the seed medium about 5.77) per 50 mL of fermentation medium, respectively.

### 3.2. Mixture Design for Optimizing the Proportion of Different Carbon Sources

**Figure 2:**
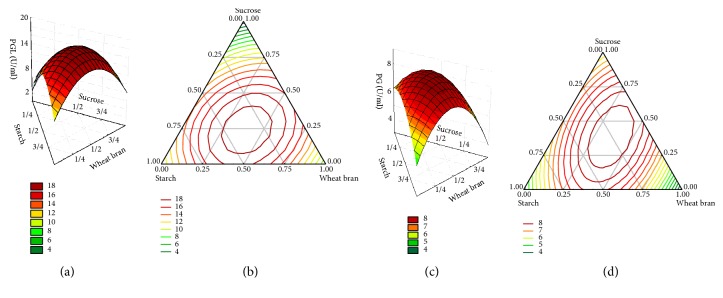
Response surfaces and contour plots for PGL (a, b) and PG (c, d) production in the mixture design experiments.

Many carbon sources have been used in submerged fermentation with* Bacillus *sp. Y1 to screen suitable carbon sources for pectinase production in preliminary work (data not shown). It was found that three carbon sources, namely, starch, wheat bran, and sucrose, were suitable for producing pectinase using* Bacillus *sp. Y1. In the present study, their proportion in the mixed carbon source was further optimized by the mixture design method. Table 1 showed the activities of the PGL and PG in crude pectinases at 72 h of fermentation, and Figure 2 displayed the response surfaces and contour plots of the carbon sources on PGL and PG activity. The optimal proportion of the three carbon sources in the mixture for the PGL and PG production was determined using the Minitab statistical software, which was 0.36, 0.40, and 0.24 for starch, wheat bran, and sucrose, respectively. The quadratic model of response Y and independent variables were expressed as follows:

(3) PGLU/mL=10.072Xa+7.977Xb+2.529Xc+32.782XaXb+28.957XaXc+42.920XbXc

(4) PGU/mL=5.142Xa+3.862Xb+6.268Xc+13.207XaXb+5.187XaXc+11.928XbXc

The* F*-value and* P*-value of the PGL model were 7.80 and 0.034 (*P* < 0.05) and 11.14 and 0.018 (*P* < 0.05) for the PG model, so the estimated models fit the experimental data adequately. The coefficient of determination* R*^*2*^ was 0.9070 and 0.9330 for the PGL and PG model, respectively, indicating that the model was able to comprehend a 90% of data variability.

A verification experiment was conducted to evaluate the reasonability of the proportions, in which the three substrates were mixed according to the proportions and were used as carbon sources in submerged fermentation of* Bacillus *sp. Y1. It was proved that the pectinases from* Bacillus *sp. Y1 using the mixed carbon sources had higher activities of PGL and PG (19.7 and 10.1 U/mL, respectively) than that using a single carbon source (No. 1, 7, and 9 in Table 1). Therefore, compared to the initial components of carbon source used in experiment, the carbon source mixture composed of 36% starch, 40% wheat bran, and 24% sucrose was more suitable for pectinase production of* Bacillus *sp. Y1.

### 3.3. Screening of Significant Factors that Affect Pectinase Production in Culture Media

Some factors such as carbon and nitrogen sources, phosphorus, and surfactant affected enzyme production too. In this study, the FFD method was used to screen the factors that significantly affect pectinase production. Factors and levels used in the FFD, the experimental design and results were shown in Tables 2 and 3, respectively. The results were analyzed using standard ANOVA. The model equations of fermentation could be written as follows:

(5) PGLU/mL=11.519−0.623x1−3.123x2−0.167x3+0.684x4−0.545x5−3.522x6+0.586x1x2+0.515x1x3+0.271x1x4+0.222x1x5−1.180x1x6−0.389x2x4−2.389x2x6

(6) PGU/mL=11.477+0.615x1−2.203x2+0.099x3+0.431x4−0.259x5−3.705x6−0.454x1x2−0.066x1x3+0.450x1x4−0.197x1x5−2.140x1x6−0.039x2x4−1.687x2x6

The* F-* and* P*-values for the PGL model were 53.05 and 0.019 (*P *< 0.05) and 54.85 and 0.018 for the PG model (*P* < 0.05). These results suggested that the regression models sufficiently reflected the experimental data. The coefficient of determination (*R*^2^) was calculated to evaluate the conformance of the model with the experimental data. The closer the* R*^2^ value is to 1, the better the model is in terms of variability of the experimental data to the predicted values [18]. The* R*^2^ was 0.9953 for the PGL model and 0.9957 for the PG model. The results indicated good agreement between the predicted values and the experimental data.

Analysis of the significance (*P*-value) demonstrated that (NH_4_)_2_SO_4_ (x_2_,* P* = 0.007 < 0.01) and initial pH (x_6_,* P* = 0.006 < 0.01) were the most significant factors for pectinase production. Moreover, the effect of the interactions of x_1_x_6_ (*P* = 0.049 < 0.05) and x_2_x_6_ (*P* = 0.013 < 0.05) was significant for PGL production. For the PG activity of pectinase, initial pH (x_6_,* P* = 0.004 < 0.01) was the most significant factor. The effects of (NH_4_)_2_SO_4_ (x_2_,* P* = 0.012 < 0.05), x_1_x_6_ (*P* = 0.013 < 0.05), and x_2_x_6_ (*P* = 0.020 < 0.05) were also significant. However, other factors such as carbon source (x_1_), K_2_HPO_4_ (x_3_), MgSO_4_ (x_4_), Tween-80 (x_5_), and the interactions of x_1_x_2_, x_1_x_3_, x_1_x_4_, x_1_x_5_, and x_2_x_4_ did not significantly affect activities of PGL and PG of the pectinase (their* P*-values are not shown). Based on the results, (NH_4_)_2_SO_4_ (x_2_), initial pH (x_6_), and carbon source (x_1_) were further optimized using the central composite design to obtain their optimum levels.

### 3.4. Central Composite Design for Optimizing the Significant Factors

Table 4 showed the experimental design and results at 72 h of fermentation using the CCD method. The second-order polynomial equations for PGL and PG production were acquired through the regression analysis of results using the design expert software. The production models were as follows:

(7) PGLU/mL=26.887+0.612x1+4.534x2−2.481x6−0.917x12−3.638x22−2.577x62−1.131x1x2−0.653x1x6+2.791x2x6

(8) PGU/mL=33.733+1.148x1+3.963x2−2.685x6+0.080x12−2.958x22−2.382x62+0.927x1x2+0.968x1x6+1.714x2x6

Based on the ANOVA results, the models were highly significant for production of PGL (*F* = 29.72,* P* < 0.001) and PG (*F* = 131.23,* P* < 0.001). The* R*^2^ of the models were 0.9640 for PGL and 0.9916 for PG, which indicated that the predicted values and the experimental values were in good accordance.

Each variable coefficient was predicted through regression analysis and the significance of each coefficient was determined using the* T-* and* P*-values. Larger* T*-values and smaller* P*-values indicated that the corresponding coefficient had a highly significant effect [18]. (NH_4_)_2_SO_4_ (x_2_), initial pH (x_6_), x_2_x_2_, and x_6_x_6_ had the most significant effects (*P* < 0.001), x_2_x_6_ (*P* = 0.001 < 0.05) had a significant effect, and carbon source (x_1_) (*P* = 0.195 > 0.1), x_1_x_1_ (*P* = 0.058 > 0.05), x_1_x_2_ (*P* = 0.078 > 0.05), and x_1_x_6_ (*P* = 0.284 > 0.1) had no significant effects on PGL activity in the crude pectinase produced by submerged fermentation of* Bacillus *sp. Y1. Meanwhile, x_6_ had a negative coefficient, which indicated that lowering its level could enhance PGL production. The quadratic and interaction terms of all the three variables had negative coefficients except for x_2_x_6_. Positive effects were observed for x_1_ and x_2_, which indicated a linear effect on PGL production. For PG production, carbon source (x_1_), (NH_4_)_2_SO_4_ (x_2_), initial pH (x_6_), x_2_x_2_, x_6_x_6_, and x_2_x_6_ had the most significant effects (*P* < 0.001), x_1_x_2_ (*P* = 0.004 < 0.05), and x_1_x_6_ (*P* = 0.003 < 0.05) had significant effects, and x_1_x_1_ (*P* = 0.669 > 0.1) had no significant effect. Similarly, initial pH (x_6_) also had a negative coefficient, which indicated that decrease of initial pH may increase PG activity of crude pectinase. The quadratic terms x_2_ and x_6_ had negative coefficients. Positive effects were observed for x_1_, x_2_, x_1_^2^, x_1_x_2_, x_1_x_6_, and x_2_x_6_, which indicated a quadratic effect on PG production.

**Figure 3:**
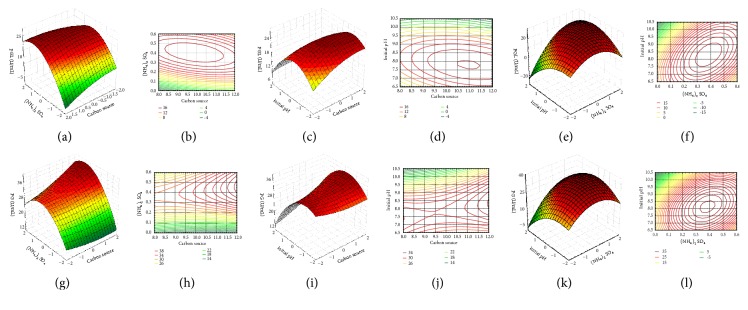
The response surface and contour plots showing relative effect of two parameters on PGL (a-f) and PG production (g-l) while other at constant level. The X-axis and Y-axis showed the value (%, w/v) of parameter and the Z-axis showed the PGL and PG activity (U/mL).

The graphical representation of the regression equation, the 3D surface plots and 2D contour plots enable the visualization of the relationship between the response and experimental levels of each variable and the type of interaction between the variables to deduce the optimum conditions (Figure 3) [18]. Elliptical contours were obtained when there was a perfect interaction between the independent variables [19]. A computation of the optimal level for each variable could be procured via these plots. We also used numerical technique with the Minitab statistical software to obtain the accurate optimal values of the variables for both PGL and PG production. The accurate optimal values for the variables were as follows: carbon source of 10.5%, (NH_4_)_2_SO_4_ of 0.37% (w/v), and initial pH 8.2.

### 3.5. Verification Experiments for Pectinase Production under the Optimized Conditions

**Figure 4:**
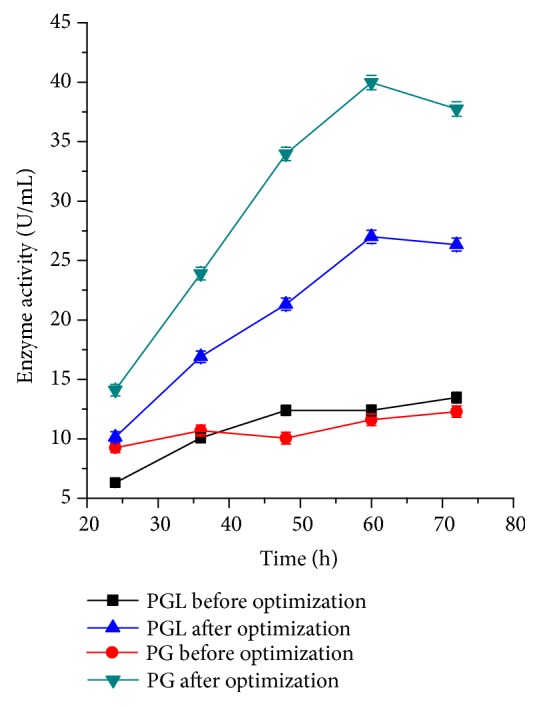
PGL and PG productions in the submerged fermentation of* Bacillus *sp. Y1 before and after the optimization of medium composition and culture conditions.

Based on the above results, verification experiments were conducted using optimized media compositions and culture conditions for pectinase production.  Figure 4 showed that the pectinase activity reached its peak at 60 h of fermentation time after optimization, that is to say, the fermentation time shortens 12 h. Moreover, PGL and PG activity (27.0 and 40.0 U/mL, respectively) increased to 2.00-fold and 3.44-fold, respectively, compared with the control (before optimization). Therefore, the final optimal medium components and fermentation conditions were as follows: carbon source, 10.5% (consisting of 3.8% starch, 4.2% wheat bran, and 2.5% sucrose); (NH_4_)_2_SO_4_, 0.37%; MgSO_4_, 0.3%; Tween-80, 0.1% (w/v); initial pH, 8.2; and inoculation amount of 1.3 mL (with the OD600 of the seed medium about 5.77) per 50 mL of fermentation medium on a swing shaker (100 rpm) at 34°C for 60 h.

## 4. Discussion

A number of the genus* Bacillus* and related genera are known to produce extracellular pectinase, which have been applied in ramie bast fibers industry [3, 8, 15]. Even though some studies about enzymatic degumming and pectinase production had already been done, more effective degumming enzymes are still needed to boost the application of enzymatic degumming technology in the industry [4, 8]. In our previous study, it was found that the alkaline pectinase produced from wild-type* Bacillus *sp. Y1 had more effective enzyme system to fast and forceful remove gum of ramie fiber. For example, the gum loss reached to 39.7% (the residual gum was 14.5%) after treatment of 2 h with lower PGL dosage (40 U PGL per gram of ramie fibers) [16]. The pectinase had also the high tolerance for H_2_O_2_. What is more, the synergistic action was also found between it and H_2_O_2_ on the degumming and bleaching of ramie fibers. The protease component in the alkaline pectinase from* Bacillus *sp. Y1 was further substantiated to be an important factor for its high degumming ability, and the synergistic action between protease and pectinase on degumming was also proved. These studies showed that the alkaline pectinase of* Bacillus *sp. Y1 had unique advantages and deserved further study.

For further enhancing the enzyme activity of* Bacillus *sp. Y1, statistical approaches were employed to optimize components of media. Our preliminary study about the effect of different carbon sources on the pectinase production (data not shown) had shown that starch, wheat bran, and sucrose were good carbon source for producing pectinase by the* Bacillus *sp. Y1. In the study, an appropriate ratio of the three carbon sources was obtained by mixture design strategy, and higher PGL and PG activity were obtained by using the optimized carbon source mixtures compared to any a single carbon source. Martins et al. reported that* Thermoascus aurantiacus* 179-5 could produce a maximum of 43 U/g of PG and 40,180 U/g of pectin lyase when grown on orange peel, sugar cane bagasse, and wheat bran as carbon sources under solid-state fermentation [20]. Silva et al. also found that maximum activity of PG (30 U/g) and pectin lyase (20 U/g) were obtained when wheat bran and orange bagasse were used as the carbon source by* Penicillium viridicatum* Rfc3 in SSF [21]. Khan et al. found* A. niger* ATCC 16404 was hyperproducer (5.38 U/mL) of pectinase using a substrate combination of wheat bran and mosambi peel as carbon source moistened with the micronutrient solution under SSF cultivation at 65% moisture and 5.0% pectin as an additional carbon source [22]. It was also found that the mixed carbon sources not only enhanced the enzyme production, but also boosted other biological products, e.g., lysine production [23].

The conventional method of ‘‘change-one-factor-at-a-time” for optimizing conditions of enzyme production is laborious, time-consuming and may lead to unreliable results and less accurate conclusions. Response surface methodology (RSM), which includes factorial designs and regression analysis, involves full factorial search by examining simultaneous, systematic, and efficient variation of all components. It is useful for small number of variables (up to five), but impractical for a large number of variables, due to a high number of experimental runs required. Therefore, for screening more than five factors, FFD is recommended [8]. In the study, the FFD was used to identify the critical variables that possible influenced the pectinase production. It was confirmed by FFD method that carbon source, (NH_4_)_2_SO_4_, and initial pH were the most significant factors for production of both PGL and PG during submerged fermentation. It also reported that C:N ratio and pH were the major factor that influenced the pectinase production [8], and lactose, tryptone, and (NH_4_)_2_SO_4_ had also significant influence on the activity of pectinase [24]. The effects of surfactants and MgSO_4_ on pectinase production have been previously reported [9, 10]. However, Tween-80 and MgSO_4_ did not have a significant effect on the pectinase production by* Bacillus *sp. Y1.

It was reported that the PG production by* Bacillus pumilus* dcsr1 increased to 21.0 and 25.3 U/mL in shake flasks and lab fermenter, respectively, by the optimization of variables using Plackett–Burman design (PB) and RSM [8]. The enhancement from 9.0 to 13.7 U/mg DW/mL in PG production of* Kluyveromyces wickerhamii* was achieved after pH, temperature, and incubation period were optimized via RSM [25]. By optimization using RSM, PGL production increased to 2.68 U/mL, 1.4-fold increase compared to the production before optimization, during submerged fermentation of* Debaryomyces nepalensis* [6], and further increased to the maximum PGL activity of 8.73 U/mL, 2.9-fold increase, after optimization via PB design and RSM [6]. By optimization of fermentation conditions using PB and RSM tools, a 2.7-fold enhancement in the PG production was achieved with* B. subtilis* NRRL B-4219 under submerged fermentation [26]. The* P. griseoroseum* T20 strain presented an increase in PL production of more than 400 fold and an increase of at least 14-fold in PG production in submerged fermentation after optimization via RSM [27]. Based on the results of FFD experiments, we used RSM to optimize these critical parameters in this study. The predicted and experimental values were very close, which all reflected the accuracy and applicability of RSM. According to the model, the production of pectinase of* Bacillus *sp. Y1 was affected by the interaction of carbon source with (NH_4_)_2_SO_4_ and initial pH. Compared to pectinase production in unoptimized medium, a 2.00-fold and 3.44-fold in PGL and PG production (27.0 and 40.0 U/mL, respectively) were achieved by submerged fermentation using the optimized medium in shake flasks. Moreover, the fermentation period at maximum pectinase production was shortened from 72 h before optimization to 60 h after optimization (Figure 4). In our previous study, the gene that encodes PGL (*pels*) has also been homologously overexpressed in* Bacillus subtilis* to enhance the PGL production [14]. In further work, we will further increase pectinase activity of* Bacillus *sp. Y1 using genetic engineering method.

## 5. Conclusions

By mixture design, FFD, CCD, and single factor experiments, the final optimal medium components and fermentation conditions for pectinase production of* Bacillus *sp. Y1 were determined as follows: carbon source, 10.5% (consisting of 3.8% starch, 4.2% wheat bran, and 2.5% sucrose); (NH_4_)_2_SO_4_, 0.37%; MgSO_4_, 0.3%; Tween-80, 0.1% (w/v); initial pH, 8.2; and inoculation amount of 1.3 mL (with the OD600 of the seed medium about 5.77) per 50 mL of fermentation medium on a swing shaker (100 rpm) at 34°C for 60 h. After optimization, PGL and PG activity (27.0 and 40.0 U/mL, respectively) increased to twofold and 3.44-fold, respectively, and the fermentation time shortened 12 hours (from 72 h to 60 h). The results are valuable for pectinase production of* Bacillus *sp. Y1, reducing production cost, and providing a basis for further research, such as genetic means to further increase enzyme activity.

## Figures and Tables

**Table 1:** Experimental design and pectinase activities (U/mL) of the mixture design.

Run no.	Starch		Wheat bran		Sucrose		PGL		PG	
	ratio	actual value (g/L)	ratio	actual value (g/L)	ratio	actual value (g/L)	Experiment	Predicted	Experiment	Predicted
1	0	0.0	0	0.0	1	70.0	3.4 ± 0.2	2.5	6.4 ± 0.3	6.3
2	1/6	11.7	2/3	46.7	1/6	11.7	18.1 ± 0.3	16.6	7.2 ± 0.3	7.4
3	1/2	35.0	1/2	35.0	0	0.0	17.4 ± 0.3	17.2	8.1 ± 0.3	7.8
4	2/3	46.7	1/6	11.7	1/6	11.7	16.4 ± 0.2	16.5	7.0 ± 0.2	7.5
5	1/2	35.0	0	0.0	1/2	35.0	15.4 ± 0.2	13.5	7.4 ± 0.3	7.0
6	1/6	11.7	1/6	11.7	2/3	46.7	9.9 ± 0.3	13.6	7.2 ± 0.2	7.9
7	0	0.0	1	70.0	0	0.0	7.1 ± 0.2	8.0	3.8 ± 0.2	3.9
8	1/3	23.3	1/3	23.3	1/3	23.3	17.8 ± 0.4	18.5	8.7 ± 0.4	8.5
9	1	70.0	0	0.0	0	0.0	9.8 ± 0.3	10.1	5.2 ± 0.2	5.1
10	0	0.0	1/2	35.0	1/2	35.0	17.3 ± 0.3	16.0	8.4 ± 0.3	8.0

**Table 2:** Factors and levels in the fractional factorial design.

Factors	Level of factors (g/L)		
	–1	0	+1
Carbon source (*x*_1_)	50.0	70.0	90.0
(NH_4_)_2_SO_4_ (*x*_2_)	3.0	8.0	13.0
K_2_HPO_4_ (*x*_3_)	0.0	0.5	1.0
MgSO_4_ (*x*_4_)	1.0	2.0	3.0
Tween-80 (*x*_5_)	1.0	2.0	3.0
Initial pH (*x*_6_)	7.5	8.5	9.5

**Table 3:** Experimental design and pectinase activities (U/mL) using the fractional factorial design method.

Run no.	Carbon source	(NH_4_)_2_SO_4_	K_2_HPO_4_	MgSO_4_	Tween-80	Initial pH	PGL		PG	
							Experiment	Predicted	Experiment	Predicted
1	–1	–1	–1	1	–1	1	17.6 ± 0.7	18.2	12.3 ± 0.6	12.7
2	–1	–1	1	–1	1	1	13.5 ± 0.5	13.6	12.4 ± 0.7	12.8
3	–1	–1	1	1	1	–1	15.3 ± 0.3	15.2	13.0 ± 0.6	12.6
4	–1	1	1	1	–1	1	4.3 ± 0.2	3.8	6.3 ± 0.3	6.0
5	1	–1	1	–1	–1	1	10.6 ± 0.3	10.4	10.5 ± 0.4	10.2
6	1	1	1	–1	1	–1	15.4 ± 0.6	14.9	16.0 ± 0.8	15.7
7	1	–1	1	1	–1	–1	17.6 ± 0.9	17.8	19.9 ± 0.9	20.3
8	1	1	–1	1	–1	–1	16.1 ± 0.8	16.0	18.6 ± 1.0	18.2
9	–1	–1	–1	–1	–1	–1	17.0 ± 0.8	16.4	12.7 ± 0.6	12.4
10	1	1	–1	–1	–1	1	0.5 ± 0.0	0.7	1.1 ± 0.1	1.5
11	–1	1	1	–1	–1	–1	12.7 ± 0.4	13.2	12.3 ± 0.6	12.6
12	1	–1	–1	1	1	1	12.3 ± 0.5	11.8	11.3 ± 0.5	11.0
13	1	1	1	1	1	1	1.3 ± 0.2	1.9	2.0 ± 0.1	2.3
14	1	–1	–1	–1	1	–1	13.2 ± 0.4	13.7	17.2 ± 0.8	17.5
15	–1	1	–1	–1	1	1	3.7 ± 0.1	3.6	6.1 ± 0.2	5.7
16	–1	1	–1	1	1	–1	13.0 ± 0.7	13.1	11.7 ± 0.5	12.1
17	0	0	0	0	0	0	13.4 ± 0.8	11.5	16.6 ± 0.9	11.5
18	0	0	0	0	0	0	12.4 ± 0.6	11.5	13.5 ± 0.4	11.5
19	0	0	0	0	0	0	14.3 ± 0.3	11.5	14.2 ± 0.3	11.5

**Table 4:** Experimental design and pectinase activities (U/mL) of the central composite design.

Run no.	Experimental factors and code levels			PGL		PG	
	Carbon source	(NH_4_)_2_SO_4_	Initial pH	Experiment	Predicted	Experiment	Predicted
	(%, v/w)	(%, v/w)					
1	–1 (9.00*∗*)	+1 (0.45)	–1 (7.50)	22.1 ± 0.6	23.8	31.9 ± 0.8	32.3
2	–1.68 (8.32)	0 (0.30)	0 (8.50)	23.2 ± 0.9	23.3	31.7 ± 1.0	32.0
3	0 (10.00)	0 (0.30)	0 (8.50)	26.9 ± 1.1	26.9	33.8 ± 1.4	33.7
4	+1 (11.00)	+1 (0.45)	+1 (9.50)	21.8 ± 0.7	23.4	34.7 ± 0.9	34.5
5	0 (10.00)	0 (0.30)	0 (8.50)	26.9 ± 1.1	26.9	33.8 ± 1.4	33.7
6	0 (10.00)	0 (0.30)	0 (8.50)	26.9 ± 1.1	26.9	33.8 ± 1.4	33.7
7	0 (10.00)	0 (0.30)	0 (8.50)	26.9 ± 1.1	26.9	33.8 ± 1.4	33.7
8	0 (10.00)	–1.68 (0.05)	0 (8.50)	6.5 ± 0.3	9.0	17.7 ± 1.0	18.7
9	0 (10.00)	0 (0.30)	–1.68 (6.82)	23.2 ± 1.0	23.8	31.6 ± 0.9	31.5
10	+1.68 (11.68)	0 (0.30)	0 (8.50)	24.6 ± 0.5	25.3	35.3 ± 0.4	35.9
11	0 (10.00)	0 (0.30)	0 (8.50)	26.9 ± 1.1	26.9	33.8 ± 1.4	33.7
12	0 (10.00)	+1.68 (0.55)	0 (8.50)	26.0 ± 0.5	24.2	32.1 ± 1.1	32.0
13	+1 (11.00)	+1 (0.45)	–1 (7.50)	24.8 ± 0.8	24.1	34.7 ± 0.7	34.5
14	+1 (11.00)	–1 (0.15)	+1 (9.50)	13.3 ± 0.6	11.0	22.4 ± 1.1	21.3
15	+1 (11.00)	–1 (0.15)	–1 (7.50)	23.2 ± 0.9	22.9	28.3 ± 0.6	28.2
16	–1 (9.00)	+1 (0.45)	+1 (9.50)	26.0 ± 0.9	25.8	28.9 ± 0.8	28.4
17	0 (10.00)	0 (0.30)	0 (8.50)	26.9 ± 1.1	26.9	33.8 ± 1.4	33.7
18	0 (10.00)	0 (0.30)	+1.68 (10.18)	15.2 ± 0.6	15.4	21.4 ± 1.2	22.5
19	–1 (9.00)	–1 (0.15)	+1 (9.50)	8.7 ± 0.3	8.9	19.4 ± 0.3	18.9
20	–1 (9.00)	–1 (0.15)	–1 (7.50)	20.3 ± 0.4	18.1	30.1 ± 0.6	29.7

*∗*All figures in parenthesis are the actual value of factors in the experiments.

## Data Availability

The data and materials supporting the conclusions of this article are included within the article.
